# Computer vision-based personal identification using 2D maximum intensity projection CT images

**DOI:** 10.1007/s00330-025-11630-0

**Published:** 2025-04-27

**Authors:** Andreas Heinrich, Michael Hubig, Gita Mall, Ulf Teichgräber

**Affiliations:** 1 Department of Radiology, Jena University Hospital–Friedrich Schiller University, Jena, Germany; 2 Institute of Forensic Medicine, Jena University Hospital–Friedrich Schiller University, Jena, Germany

**Keywords:** Computer vision systems, Computed tomography (X-ray), Emergency care, Human identification, Thorax

## Abstract

**Objectives:**

Computer vision (CV) mimics human vision, enabling the automatic comparison of radiological images from recent examinations with a vast image database for unique identification. This method offers significant potential in emergencies involving unknown individuals. This study assesses whether maximum intensity projection (MIP) images from thoracic computed tomography (CT) examinations are suitable for automated CV-based personal identification.

**Methods:**

The study analyzed 12,465 native CT examinations of the thorax from 8177 individuals, focusing on MIP images to assess their potential for CV-based personal identification in 300 cases. CV automatically identifies and describes features in images, which are then matched to reference images. The number of matching points was used as an indicator of identification accuracy.

**Results:**

The identification rate was 98.67% (296/300) at rank 1 and 99.67% (299/300) at rank 10, among over 8177 potential identities. Matching points were higher for images of the same individual (7.43 ± 5.83%) compared to different individuals (0.16 ± 0.14%), with 100% representing the maximum possible matching points. Reliable matching points were mainly found in the thoracic skeleton, sternum, and spine. Challenges arose when the patient was curved on the table or when medical equipment was present in the image.

**Conclusion:**

Unambiguous identification based on MIP images from thoracic CT examinations is highly reliable, even for large CV databases. This method is applicable to various 2D reconstructions, provided anatomical structures are comparably represented. Radiology offers extensive reference images for CV databases, enhancing automated personal identification in emergencies.

**Key Points:**

***Question*** *Computer vision-based personal identification holds great potential, but it remains unclear whether maximum intensity projection images from thoracic-CT scans are suitable for this purpose.*

***Findings*** *Maximum intensity projection images of the thorax are highly individual, with computer vision-based identification achieving nearly 100% rank-1 accuracy across a potential 8177 identities.*

***Clinical relevance*** *Radiology holds a vast collection of reference images for a computer vision database, enabling automated personal identification in emergency examinations. This improves patient care and communication with relatives by providing access to medical history.*

**Graphical Abstract:**

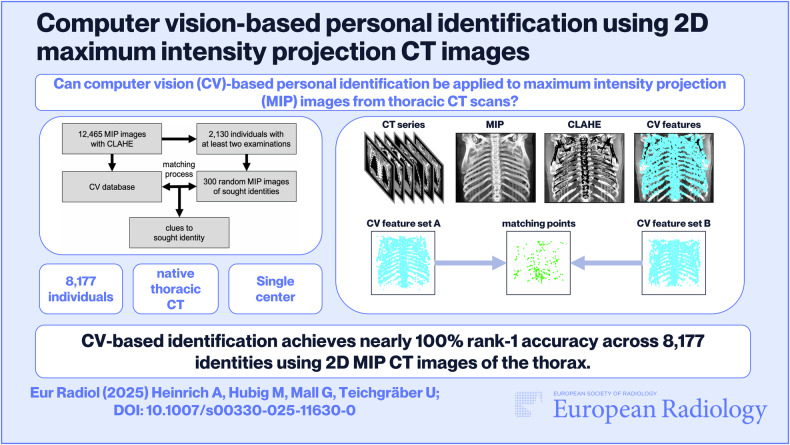

## Introduction

Natural disasters, terrorist attacks, war, severe accidents, migration, or homelessness can lead to unknown individuals being admitted to emergency rooms [1–6], where identification poses significant challenges. In such cases, computed tomography (CT) plays a crucial role, offering essential diagnostic support and serving as a non-invasive tool for detailed anatomical examination. Further utilization of CT data for automated identification of unknown individuals could be novel and particularly valuable in time-critical situations.

A novel computer vision (CV)-based personal identification method has successfully identified individuals using single CT slices by matching a cranial CT image of an unknown person with a large CV database [7]. CV mimics human vision by automatically identifying unique features in images. These CV features can then be recognized between a query image of an unknown individual and a reference image, resulting in so-called matching points. More matching points indicate more probable correct identification of the individual. However, a key limitation of this method is its reliance on selected axial slices, which are susceptible to variations in head orientation and metallic artifacts, increasing measurement uncertainty.

This study aims to explore whether single CT slices of the thorax, using maximum intensity projection (MIP) images, can simplify and expand the identification process with the CV-based personal identification method. Existing literature suggests that the thoracic skeleton, sternum, and spine display distinctive features that can assist in identification by forensic experts, as demonstrated in two case reports [8, 9].

## Materials and methods

The study was approved by the local institutional review board (IRB) at Jena University Hospital (registration number 2019-1505-MV). Due to the retrospective nature of the investigation, the IRB waived the requirement for written informed consent.

**Fig. 1:**
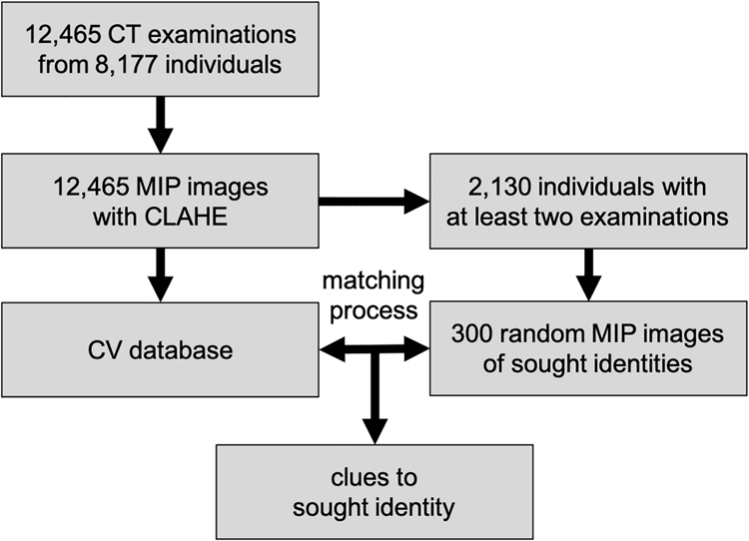
From 12,465 CT examinations consisting of coronal images, a MIP image was calculated for each, and the contrast was optimized using CLAHE. Subsequently, CV was applied, and the features detected for each examination were stored in the CV database. A total of 2130 individuals had at least two MIP images, and for 300 individuals, a random recent MIP image was selected to match against the CV database. If the result with the highest score (most matching points = best result) corresponds to the sought individual, the identification is considered successful

This retrospective study analyzed 12,465 consecutive native thoracic CT examinations from 8177 individuals (ages 8–102 years, mean age 65.13 ± 14.03 years; see Fig. 1 and Table 1) conducted between November 2015 and February 2024 in the emergency department and clinical routine using two GE Revolution scanners (voltage 70–140 kVp, current 232.00 ± 109.29 mA). Each examination included coronal thoracic images in soft tissue settings with a slice thickness of 4.83 ± 0.56 mm. No exclusion criteria were applied.

**Table 1:** Demographics table of study participants from 12,465 native CT examinations, which were added to the CV database

Age range (years)	Antemortem CV database			
	All	Female	Male	Unknown
0–9	5	3	2	0
10–19	43	2	37	4
20–29	197	47	137	13
30–39	451	228	194	29
40–49	800	479	269	52
50–59	2310	1470	739	101
60–69	3630	1134	2330	166
70–79	3195	1099	1907	189
80–89	1641	898	583	160
90–99	190	79	67	44
100–109	3	1	2	0
All	12,465	4150	7557	758

### Signal processing, CV feature extraction and matching process

**Fig. 2:**
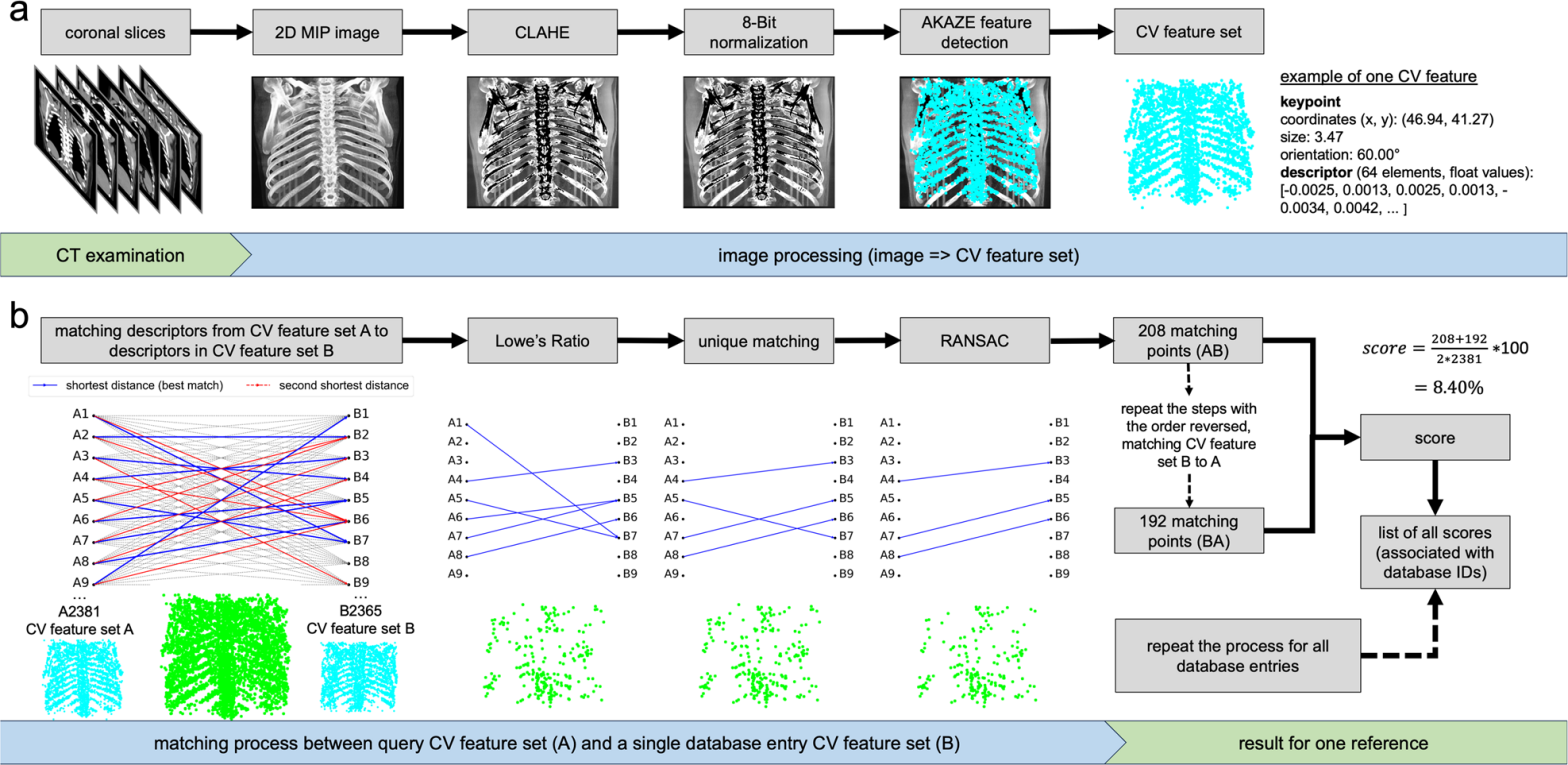
The figure illustrates the processing steps for CV-based personal identification. **a** CV feature extraction: A 2D MIP image is generated from a CT series with coronal slices. The MIP image is enhanced using CLAHE and normalized to 8-bit. CV features are then detected using the AKAZE algorithm, producing a CV feature set. This CV feature set can be independently utilized (without image data), such as by storing it as an entry in a CV database. Each CV feature comprises a keypoint and its corresponding descriptor. **b** Matching process: In the matching process, each descriptor from the query CV feature set (A) is compared to all descriptors in the reference CV feature set (B) of a single database entry based on the measurement of squared Euclidean distance. For each descriptor in A, the closest match (shortest distance) and the second-closest match in B are determined. Each descriptor in A is linked to its closest match in B, while not all descriptors in B need to be assigned to A. A single descriptor in B can be associated with multiple descriptors in A. Lowe’s ratio test is then applied by comparing the distance between the closest and the second-closest match. Only those matches with a sufficiently low ratio are kept, ensuring that the most reliable matches are retained. Furthermore, each descriptor in B can be assigned to at most one descriptor in A to ensure a unique matching, where only the best result (closest match) is kept. Finally, RANSAC is used to remove outliers by iteratively selecting random subsets of matches and refining the transformation, ensuring that only the most consistent matches remain, which enhances the overall robustness of the matching process. At the end, a number of matching points is obtained. This process is then repeated with the order of the CV feature sets reversed. From both runs, a score is calculated by dividing the average number of matching points by the total number of CV features in set A

Coronal images were processed by creating a 3D volume from all slices of the CT series. For each pixel in this volume, the highest value was selected to produce a 2D MIP image (see Fig. 2a). Contrast-limited adaptive histogram equalization (CLAHE) was then applied to enhance the image contrast, especially in regions that were too dark or too bright. CLAHE works by dividing the image into smaller blocks and applying histogram equalization locally within each block. The effectiveness of CLAHE is influenced by two parameters:

- clipLimit: Controls the maximum amount of contrast enhancement applied locally by limiting histogram adjustment.
- tileGridSize: Defines the size of the tiles used for local histogram equalization. Smaller tiles allow for more detailed contrast adjustments.

After normalizing the MIP image to 8-bit color depth, the AKAZE algorithm [10, 11], implemented using OpenCV 4.5.3, was applied to detect robust CV features. AKAZE is an image feature extraction algorithm based on nonlinear differential equations. It first transforms the image into a scale-space representation by creating octaves, each containing images at different resolutions. Within each octave, layers are formed that represent various levels of image detail at different scales. This allows AKAZE to detect stable features across different levels of image resolution. The algorithm reduces noise using nonlinear diffusion filtering, which smooths out irrelevant parts while preserving important details like edges. To find keypoints, AKAZE uses the Hessian matrix (second derivatives of image intensity) to identify extrema—points where the image intensity changes significantly, such as edges or corners. For each keypoint, AKAZE computes a descriptor, a “fingerprint” that captures the local structure around the keypoint. This descriptor is rotation- and scale-invariant, making it robust to image transformations like rotation or resizing. It is abstract and does not allow for image reconstruction, but it captures the essential features of a keypoint’s local structure, enabling it to be recognized in different images. In summary, a CV feature consists of a keypoint and its associated descriptor. Keypoints are distinctive points in the image, while descriptors summarize the local area around them. Five parameters govern CV feature extraction:

- Octaves: Determines the number of times the image is scaled down to detect features at different sizes.
- Layers: Indicates the number of detail levels examined within each octave.
- Diffusivity: Determines how the algorithm processes edges and textures during the feature detection process (PM_G1: Moderate contrast, PM_G2: Stronger contrast, WEICKERT: Gentle contrast, preserves details, CHARBONNIER: Nonlinear, better edge preservation).
- Descriptor Type: Defines the type of descriptors used (KAZE: great for multi-angle features, MLDB: efficient binary pattern representation, Upright: non-rotation-invariant descriptors).
- Threshold: Determines the minimum value of the Hessian matrix determinant required for a point to be considered a valid keypoint. This threshold filters out unstable points, retaining only those with strong, distinct features.

Detected CV feature sets, along with metadata (study date, birth date, sex, and patient ID) from the digital imaging and communications in medicine (DICOM) header, were stored in an encrypted CV database. During the matching process (see Fig. 2b), the CV feature set from an unknown individual is compared to those in the CV database. For each descriptor in the query CV feature set, the two most similar descriptors in the reference set (database entry) are identified using squared Euclidean distance. Subsequently, it applies Lowe’s test to ensure that only reliable matches are retained, thereby ensuring that the distance to the closest neighbor (most similar descriptor) is significantly smaller than to the second-closest neighbor. Each CV feature has at most one corresponding match to ensure unique matching points. The random sample consensus (RANSAC) algorithm is then used to further enhance accuracy by eliminating outlier matching points. RANSAC tests various scenarios to determine the optimal model fit, ensuring that misleading matching points are excluded. The matching process involves two key parameters that affect the final number of matching points:

- Lowe’s Ratio: Sets the threshold ratio for Lowe’s test. For example, with a ratio of 0.5, the best match must be at least twice as close as the second-best match.
- RANSAC: Defines the threshold for distinguishing inliers from outliers by using an iterative method to estimate model parameters, improving accuracy by excluding outliers.

The final similarity score, ranging from 0% (no match) to 100% (identical images), is calculated by normalizing the number of matching points against the total number of CV features in the query image:

$${\mathrm{Score}}={{\rm{matching}}}\; {{\rm{points}}}/{{\rm{total}}}\; {{\rm{CV}}}\; {{\rm{features}}}\,[ \% ]$$

For example, if the query set has 1000 CV features and yields 50 matching points, the score is 5%. The matching process was performed twice: first by comparing the query set (A) to a database entry (B), and then the reverse (B to A). The final score was calculated as the average number of matching points from both directions, ensuring a more robust comparison by considering both orientations.

### Parameter optimization and evaluation

To determine the optimal parameters, a systematic variation of settings was conducted for MIP images of 70 randomly selected identities (35 female, 35 male) across seven age categories (20–89 years, 5 males and 5 females per decade). Starting with the initial parameter settings listed in Table 2, the clipLimit was adjusted from 0 to 150. Following this, the tileGridSize was varied from 2 to 32 based on the optimal clipLimit. The octaves parameter was then adjusted from 3 to 10 using the optimal values for tileGridSize and clipLimit. Initial settings for AKAZE were derived from the publication [7], though this study did not include additional edge enhancement using a modified Sobel gradient.

**Table 2:** Overview of the parameters of the CV-based identification method and their systematic variation to determine the optimal settings

Variable	Variation [step size]	Start value	Best value
clipLimit	0–150 [10]	-	100
tileGridSize	2, 4, 8, 16, 32	8	8
Octaves	3–10 [1]	4	4
Layers	3–10 [1]	4	4
Diffusivity	1 = PM_G1 2 = PM_G2 3 = WEICKERT 4 = CHARBONNIER	2	2
Descriptor type	1 = MLDB 2 = MLDB upright 3 = KAZE 4 = KAZE upright	3	3
Threshold	0.0005–0.0025 [0.0005]	0.001	0.001
Lowe	0.1–0.9 [0.1]	0.6	0.6
RANSAC	1–20 [1]	10	13

With a clipLimit value of 0, no CLAHE was applied

All 12,465 MIP images were used to extract CV features, which were then stored in the CV database without including the original image data (see Table 1 for details on age and sex). Of the 8177 individuals, 2310 had at least two MIP images. For 300 randomly selected individuals from this group, the most recent MIP image across six age categories (30–89 years, 25 males and 25 females per decade) was used to match against the CV database. Identical MIP images between the query and the CV database were excluded from the results.

The identification rate evaluates the accuracy of recognizing individuals. “Rank 1” indicates that the score for images of the same identity surpasses the scores for all other identities. In “rank 5” and “rank 10,” the score for the sought individual is among the top 5 or 10 scores, respectively. Statistical significance (α = 0.05) was assessed using the Mann–Whitney U test to compare score distributions between groups (same identity vs. different identities), implemented using Python’s SciPy library. Pairwise comparisons between different age categories for images of the same identities were also conducted, with *p*-values adjusted using the Bonferroni correction to account for multiple comparisons.

## Results

**Fig. 3:**
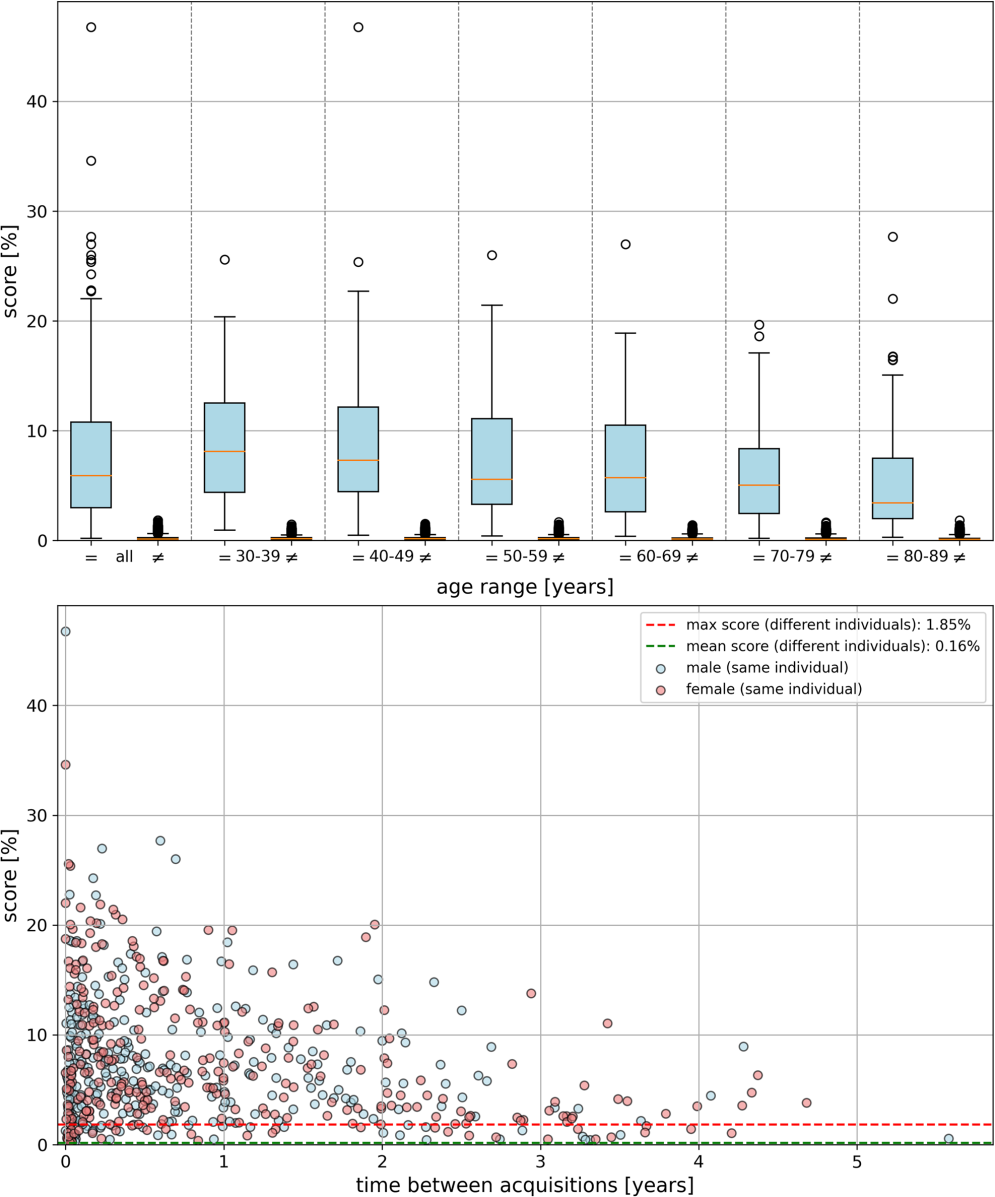
The boxplot (above) displays the results of CV-based personal identification across different age categories of the query image, illustrating comparisons between the same individual (=) and different individuals (≠). The score represents the number of matching points divided by the total number of CV features in the query image. The subfigure below illustrates the relationship between the score and the time between CT acquisitions of the same individual, demonstrating successful identification even for images taken several years apart

In this study, 12,465 CT examinations from 8177 individuals, all living patients from the emergency department and clinical routine, were analyzed to evaluate the feasibility of unique identification using MIP images of the thorax in 300 identification procedures. Overall, 296 out of 300 identities (98.67%) were correctly identified at rank 1. The remaining 4 identities were identified at ranks 2, 4, 7, and 919, resulting in identification rates of 99.33% at rank 5 and 99.67% at rank 10. Images from the same individual achieved higher matching points, with a score of 7.43 ± 5.83%, compared to images of different individuals, which scored 0.16 ± 0.14% (see Fig. 3 and Table 3). A comparison of the scores between same identity and different identities showed a *p*-value of < 0.001, indicating a highly significant difference between these groups. For the same identities, pairwise comparisons of age categories using the Mann–Whitney U test, with Bonferroni correction, revealed significant differences in scores between the 30–39 and 60–69 years (*p* = 0.001), 30–39 and 70–79 years (*p* < 0.001), 30–39 and 80–89 years (*p* < 0.001), 40–49 and 80–89 years (*p* < 0.001), and 50–59 and 80–89 years (*p* < 0.001) age groups, while no significant differences were observed between the remaining pairs.

**Table 3:** Results of CV-based personal identification

Age range (years)	Score [%]				DIFF MED	Identification rate [%]				
	MW (=)	MED (=)	MW (≠)	MED (≠)		Rank 1	Rank 5	Rank 10	Query rank 1	Global rank 1
30–39	8.77 ± 5.31	8.09	0.18 ± 0.14	0.18	7.91	98	98	100	99.14	93.10
40–49	8.67 ± 6.78	7.14	0.17 ± 0.14	0.17	6.97	100	100	100	98.11	91.51
50–59	8.00 ± 6.13	5.60	0.18 ± 0.14	0.18	5.42	100	100	100	96.97	92.93
60–69	6.70 ± 5.22	5.66	0.16 ± 0.13	0.15	5.51	98	98	98	99.11	89.29
70–79	6.40 ± 5.56	5.05	0.15 ± 0.14	0.12	4.93	100	100	100	93.33	82.67
80–89	5.58 ± 5.07	3.51	0.13 ± 0.13	0.11	3.40	96	100	100	95.05	79.21
All	7.43 ± 5.83	5.93	0.16 ± 0.14	0.14	5.79	98.67	99.33	99.67	97.21	86.86

MW denotes the mean value, and MED represents the median for the same individual (=) or different individuals (≠) of the score (number of matching points divided by the total number of CV features in the query image). DIFF MED is the difference in the median scores between the same individual and different individuals. “Query rank 1” indicates how many reference images of the searched identity scored higher than the maximum score between different identities in the same identification process. In contrast, “global rank 1” shows how many reference images of the searched identity scored above 1.85% (the highest score between different identities across all comparisons in this study)

Among all reference images of the searched identity in the CV database, 592 out of 609 images (97.21%) achieved rank 1 compared to images of different identities (see Table 3, column “query rank1”). Only 17 images from 10 identities did not achieve rank 1. Additionally, 529 out of 609 images (86.86%) had scores higher than the maximum score of 1.85%, found across over 3.7 million comparisons between different individuals (see Table 3, column “global rank1”). In 272 of 300 (90.67%) identification procedures, the maximum score for comparisons between images of the same identity was greater than 1.85%, making the identification clear enough that a full CV database search was theoretically unnecessary. Figure 3 illustrates the relationship between the score and the time between CT acquisitions for the same individual, showing successful identification even for CT examinations taken several years apart. Of the 300 identities, 161 had only one reference entry in the CV database, while 63 had 2 entries, 40 had 3 entries, 15 had 4 entries, 7 had 5 entries, and 14 had more than 5 entries.

**Fig. 4:**
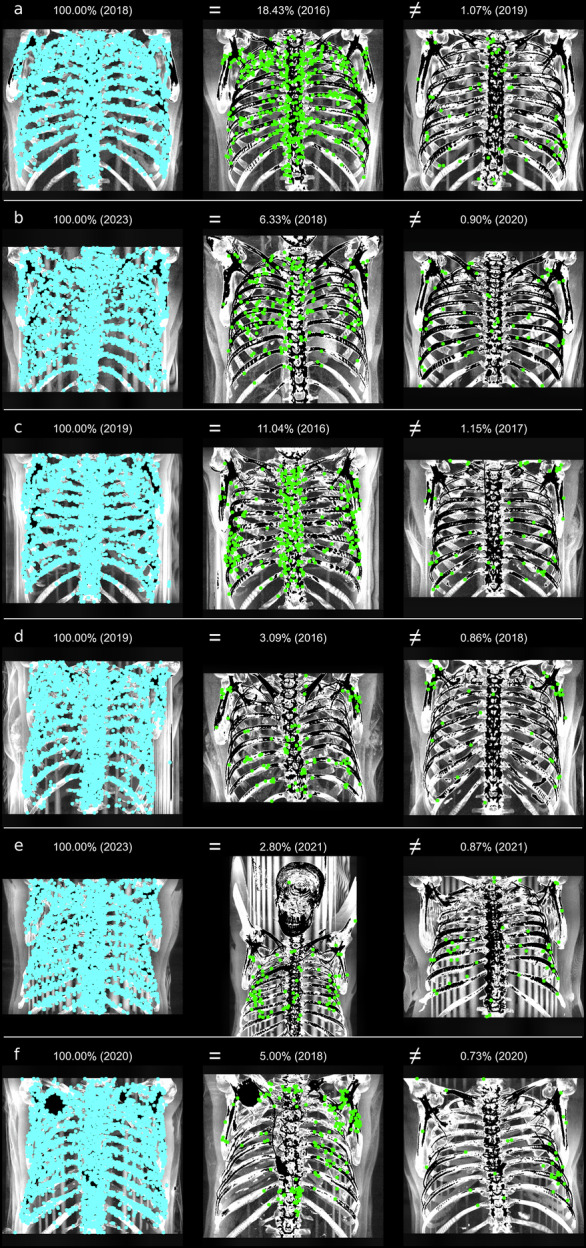
Examples of query images (left), an interesting reference image of the same identity (‘=’, middle), and the best result (highest score) for different identities (‘≠’, right), with the score indicated as a percentage and the image acquisition year in parentheses. Although structures may exhibit similarities leading to matching points, these similarities are typically much lower between different individuals compared to the same individual. The letters **a** (30–39 years) to **f** (80–89 years) correspond to the age categories of the unknown identity in the query image

Figure 4 presents examples of successful identifications. For each age category (a-f), an example (not necessarily the highest score) is shown for matching the same identity. For comparisons between different identities (right side of the image), the result with the maximum score is displayed. The thoracic skeleton, and especially the sternum and spine, has many distinct features that result in numerous matching points for the same identity, even when different CT parameters such as slice thickness or voltage are used. It is evident that successful identification can occur even if the image section varies, as long as part of the image is comparable to the query image (see Fig. 4e).

**Fig. 5:**
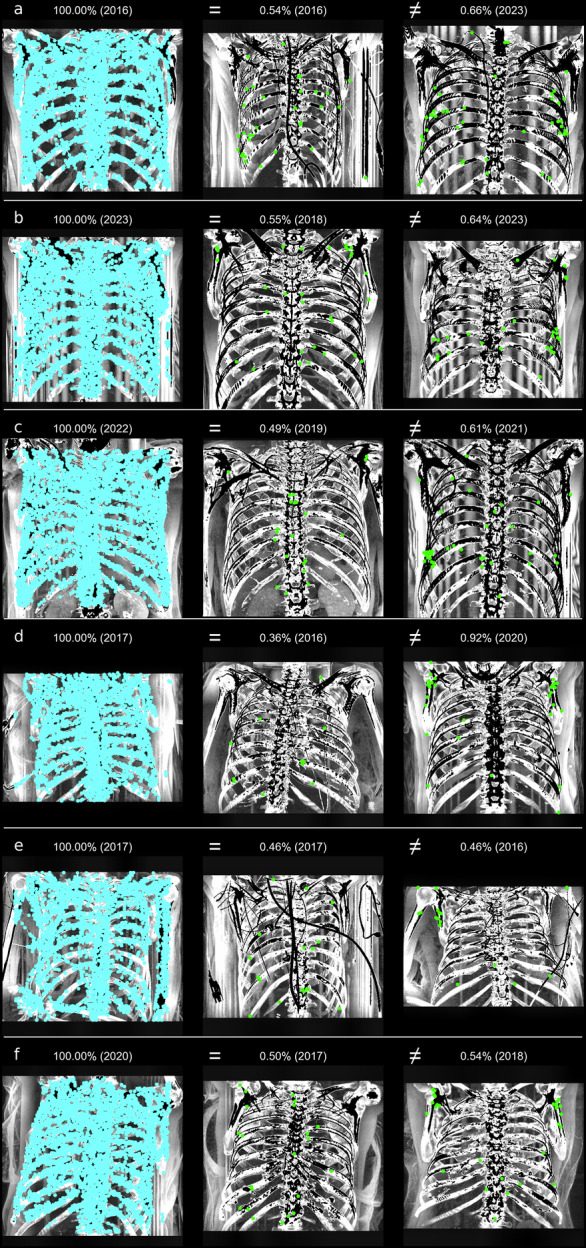
Examples of unsuccessful identifications in rank 1 of query images (left), an interesting reference image of the same identity (‘=’, middle), and the best result (highest score) for different identities (‘≠’, right), with the score indicated as a percentage and the image acquisition year in parentheses. The same identity may receive a lower score, for example, when the sternum and spine are obscured or the contrast is reduced. The letters **a** (30–39 years) to **f** (80–89 years) correspond to the age categories of the unknown person in the query image

Figure 5 shows examples of unsuccessful identifications at rank 1. Issues such as overlay by medical equipment in the search and/or reference image (a, d, e), the patient being curved on the table (d, f), or reduced contrast in the query image due to objects outside the patient, such as the CT table (b, c) resulted in significantly fewer matching points. These issues are more frequent with increasing age, which is why the score slightly decreases with age (see Fig. 3 and Table 3). However, by analyzing MIP images using slices exclusively from within the body for the query image, rank 1 identification was still achieved even in cases with reduced contrast.

**Fig. 6:**
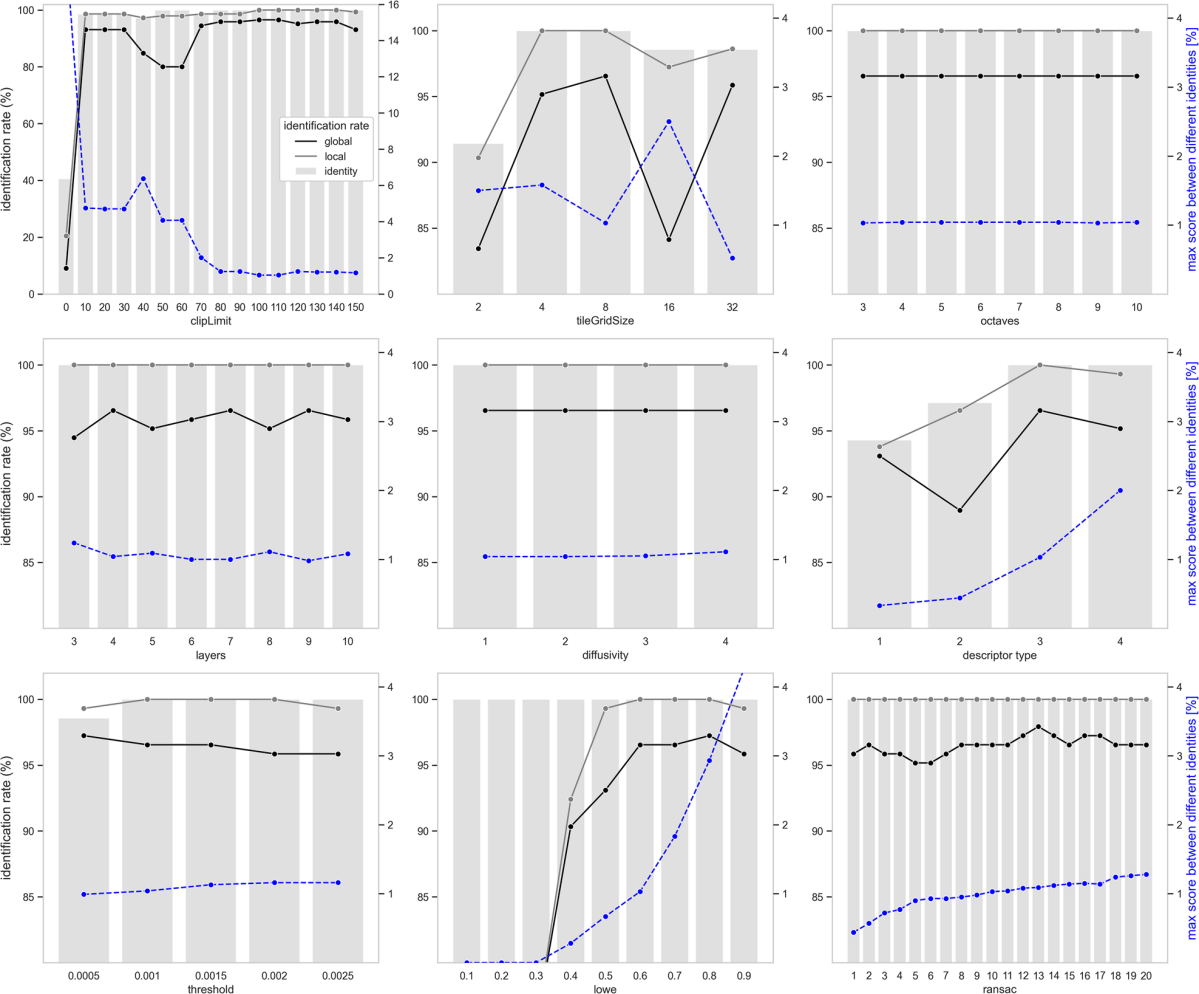
The identification rate based on systematic parameter variation (see Table 2) refers to the successful recognition of the identity at rank 1. The local identification rate means reference images of the same identity scored higher than comparisons with different identities in the identification process. The global identification rate indicates that these reference images scored higher than the maximum score found between different identities across all procedures. The maximum score between different identities, shown in blue, should be kept low, influencing parameter selection. A clipLimit of zero means CLAHE was not applied

The systematic parameter variation (see Fig. 6) demonstrates that the identification rate is significantly better with CLAHE. The best results were achieved with CLAHE and a clipLimit of 100. The initial choice of parameters for CV feature extraction and the matching process was largely confirmed (see Table 2).

## Discussion

This study demonstrates the feasibility of using CV for personal identification based on MIP images from thoracic CT examinations of living individuals, achieving nearly 100% identification rate across 300 procedures and 8177 potential identities. In CV, distinct features, such as edges and corners, are crucial, and the thoracic skeleton, sternum, and spine provide ample unique markers. While standard MIP images often lack contrast, applying CLAHE significantly improved it, leading to more matching points and higher identification rates. The CV features are robust against rotation, scale, and lighting changes, allowing reliable identification even with varying fields of view. This method can be applied to various 2D reconstructions, provided that anatomical structures are represented comparably, meaning no severe distortions, missing overlaps of identical regions, and adequate contrast.

Apart from the cranial CT study [7], no other research has applied CV methods for personal identification using CT images. The literature on thoracic CT for unique identification is limited. Ueda et al [12] compared scout CT images from two routine torso CT exams within a year to determine if they were from the same patient, using geometric correction, local feature extraction, template matching, and RANSAC. A limitation of Ueda’s method is the requirement for matching scout images, identical equipment, consistent image quality, and a short time span to avoid age-related changes. In contrast, the recent study applies CV to MIP images, identifying distinct features in the thoracic skeleton, sternum, and spine, allowing differentiation even years apart. AKAZE [10, 11] is a traditional CV algorithm that uses predefined mathematical methods to extract features based on image intensity and gradients, without involving training or data-driven learning. The parameters control how these operations are applied, but are not influenced by the data. Unlike artificial intelligence (AI)-based models, the algorithm does not adapt or “learn” from the data, meaning that optimizing parameters using the same dataset does not bias the results. The similarity of parameters to those used in study [7] on different datasets underscores AKAZE’s robustness across varied applications, with only minor adjustments needed for thoracic MIP CT images due to preprocessing differences. Furthermore, the order of image comparison can affect the matching score, as Lowe’s method and RANSAC may produce different results depending on the direction, because the relative positions and distances of keypoints can vary, influencing the selected matches and the consistency of the results. To account for this, matching is performed in both directions (AB and BA), and the final score is averaged to ensure a more reliable result.

The CV-based personal identification is fundamentally not a legally secure method for identification [13]. Its task is rather to locate suitable reference materials and/or obtain clues to the sought individual. By narrowing down the possible identity from thousands of potential identities, a legally secure and forensic identification is subsequently facilitated with appropriate reference materials by a highly qualified professional. The method relies on CV features, which cannot be used to reconstruct the original images. These features are decoupled from the image itself and, therefore, do not contain personal data. No image data is stored in the CV database. During the matching process, it can only be determined that the CV features of an unknown identity correspond with the CV features of an identity in the CV database. A pseudonymized patient ID linked to the CV feature set allows for the identification of the person. If the decryption of this ID can only be performed by the institution where the CT scan was conducted, it would ensure that data control remains with the originating facility. To address major ethical and legal concerns, one solution could be to store only CV features in central databases without personal data, including only their origin. If a high match occurs, the originating institution’s CV database, containing the patient ID, could be queried for identification. This approach mitigates risks to patient data and reduces unlawful use of sensitive information. However, like head CT scans [7, 14], sharing chest CT scans also carries a re-identification risk, although requiring access to other patient scans.

The study has some limitations, particularly regarding the selection of CT slices for MIP generation. Using all slices may reduce contrast or introduce irrelevant elements; therefore, selecting only the most relevant slices could improve the results. Additionally, incorporating filters based on sex, age, and body weight could further enhance identification accuracy [13]. Future research could explore whether severe injuries or ethnic factors affect identification accuracy, as well as compare MIP CT images with thoracic X-rays. Furthermore, the feasibility of using postmortem MIP CT images for comparison with antemortem data in the CV database to identify unidentified deceased individuals could also be investigated.

In conclusion, CV-based personal identification using MIP images from thoracic CT examinations shows high accuracy, enabling rapid identification in emergency settings. The method achieved nearly 100% correct identification across 8177 potential identities, even without additional filters. This approach is likely applicable to other 2D slices and reconstructions, further enhancing radiology’s role in providing reference images for CV-based personal identification, which could significantly improve patient care by enabling quick access to medical histories. This study paves the way for further research into the use of CV for identifying unidentified individuals through large databases in radiology.

## Abbreviations

CLAHE: Contrast-limited adaptive histogram equalization
CT: Computed tomography
CV: Computer vision
MIP: Maximum intensity projection
RANSAC: RANdom SAmple Consensus
